# Acute Hepatitis B Infection in a Patient With Confirmed Immunity on Long-Acting Cabotegravir/Rilpivirine

**DOI:** 10.14309/crj.0000000000001575

**Published:** 2024-12-27

**Authors:** William K.B. Boateng, Neil Carlin, Etan Spira, Maria E. Szabela, Kosisochukwu J. Ezeh

**Affiliations:** 1 Internal Medicine, Jersey City Medical Center, Jersey City, NJ; 2 Gastroenterology and Hepatology, Jersey City Medical Center, Jersey City, NJ; 3 Infectious Disease, Jersey City Medical Center, Jersey City, NJ; 4 Internal Medicine, Marshall University Medical Center, Huntington, WV

**Keywords:** hepatitis, hepatitis B, HIV, cabotegravir, rilpivirine

## Abstract

Long-acting injectable formulation of cabotegravir/rilpivirine (CAB/RPV) is a promising novel maintenance therapy for HIV infection. However, coinfection with active hepatitis B virus (HBV) infection is a contraindication to initiating this therapy. Despite guidelines, patients with HBV immunity can still contract acute HBV infection. We report a case of a 30-year-old man with HIV who transitioned from antiretroviral therapy to CAB/RPV and had confirmed HBV immunity. The patient, though asymptomatic, showed significantly elevated liver function tests (LFTs) before his monthly CAB/RPV injection. He was hospitalized and diagnosed with acute HBV infection. His LFTs improved, and he was taken off CAB/RPV and returned to antiretroviral therapy for the treatment of HIV and HBV. During subsequent follow-ups as an outpatient, the patient's LFTs normalized, and his HBV viral load significantly decreased. This case highlights the potential need for routine HBV testing in patients on CAB/RPV therapy.

## INTRODUCTION

The long-acting injectable formulation of cabotegravir/rilpivirine (CAB/RPV) has been a promising component in the management of HIV infection.^1^ Its efficacy and promotion of compliance with its monthly administration have been critical factors in the success of the formulation. Unlike oral antiretroviral therapies (ARTs), such as bictegravir/emtricitabine/tenofovir alafenamide (B/FTC/TAF), CAB/RPV does not have therapeutic efficacy against hepatitis B virus (HBV).^2^ Current guidelines recommend that patients who are switched to CAB/RPV cannot have an active HBV infection or must be on an HBV therapy. Despite compliance with these guidelines, patients who are known to have immunity continue to develop acute HBV while on CAB/RPV therapy.

We present a case that challenges our current understanding. Despite having immunity to HBV after vaccination, a patient with HIV developed an acute HBV infection after transitioning to CAB/RPV. This unusual turn of events prompts us to re-evaluate our approach to managing such cases.

## CASE REPORT

We present a 30-year-old man with a history of HIV who presented due to elevated liver enzymes. The patient has sex with men and was diagnosed with HIV and acquired immunodeficiency syndrome in 2013 in Ecuador when he was hospitalized for meningitis. He was initiated on an efavirenz/emtricitabine/tenofovir combination and took it until 2019. In 2019, when he came to the United States, the patient was switched to a combination of B/FTC/TAF. The patient is known to have insertive and receptive sex with multiple partners with occasional condom use. He has a history of various sexually transmitted diseases (syphilis, chlamydia, and anal herpes papillomavirus). The patient was transitioned to CAB/RPV on October 2023 from a B/FTC/TAF due to pill fatigue, despite 100 percent adherence. The patient was vaccinated for HBV before switching HIV medications. The hepatitis B surface antibody (anti-HBs) level before the change in therapy was 14.5 mIU/mL, consistent with immunity, and the hepatitis B surface antigen (HBsAg) was negative. The patient was given injectable CAB/RPV after a 1-month lead-in with oral CAB/RPV.

The patient was referred to the hospital when routine laboratory work revealed alanine transaminase (ALT) was 2876 IU/L, aspartate transaminase (AST) was 917 IU/L, and alkaline phosphatase (ALP) was 132 IU/L. All other laboratory values were within normal limits. Four months ago, the patient's ALT, AST, and ALP were within normal limits.

On presentation, the patient was asymptomatic and denied having fever, chills, scleral icterus, arthralgias, abdominal pain or tenderness, extremity swelling, and shortness of breath. The patient denied known sick contacts, recent travel, history of smoking, intravenous drug use, and sharing needles or razors. He drinks alcohol socially. He acknowledged having continued sexual encounters with multiple male partners and intermittent use of protection. The patient reported a recent piercing from a reputable piercing studio. Reported home medications were atorvastatin 10 mg and fenofibrate 48 mg, both taken daily. The physical examination was unremarkable. Vital signs were within normal limits. Laboratory findings revealed a reactive HBsAg and hepatitis B core immunoglobulin M. HBV viral load was 52,200 IU/mL. Hepatitis C antibody, hepatitis A immunoglobulin M, and a monospot test were negative. Liver function tests (LFTs) were significant for ALT 2876, AST 607, and ALP 119 IU/L. Total bilirubin, albumin, and prothrombin time and international normalized ratio were all within normal limits. Acetaminophen and alcohol levels were <2 and 6.5, respectively. The abdominal ultrasound was unremarkable.

**Figure 1.**
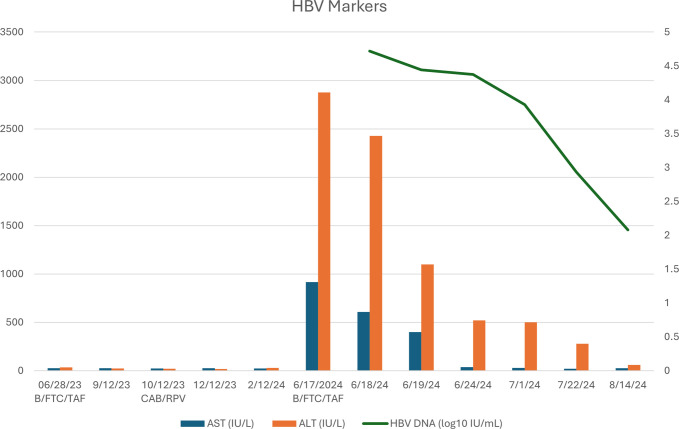
Time course of serum HBV markers over time. Solid green line, HBV DNA levels in plasma (log_10_ IU/L); orange boxes, ALT levels (IU/L); blue boxes, AST levels (IU/L). ALT, alanine aminotransferase; AST, aspartate aminotransferase; B, bictegravir; CAB, cabotegravir; FTC, emtricitabine; HBV, hepatitis B virus; TAF, tenofovir alafenamide; RPV, rilpivirine.

The patient's atorvastatin and fenofibrate were held, and the patient was kept overnight to trend LFTs. Repeat LFTs showed a decrease in ALT 1100 IU/L, AST 399 IU/L, and ALP 123 IU/L. HBV viral load decreased to 27,700 IU/mL. The patient remained asymptomatic. The patient was discharged and referred to follow-up at the infectious disease clinic the following day for repeat laboratory results. CAB/RPV was discontinued at the infectious disease clinic, and B/FTC/TAF was restarted. Repeat laboratory results showed decreased LFTs with ALT 463 IU/L, AST 38 IU/L, and ALP 99 IU/L, along with decreased HBV viral load to 8,500. The LFTs were subsequently normalized (Figure 1). The patient's partner was scheduled to be screened for HBV. The patient was educated on HBV risks and transmission.

## DISCUSSION

The long-acting injectable formulation of CAB, an integrase strand-transfer inhibitor, and RPV, a non-nucleoside reverse-transcriptase inhibitor, is approved by the US Food and Drug Administration for the treatment of HIV infection. The First Long-Acting Injectable Regimen and Long-Acting Antiretroviral Treatment Enabling Trial 2 trials have established the efficacy and safety of the formulation. Randomized trials have further concluded that the CAB/RPV combination was noninferior to oral ARTs.^1,3,4^ CAB/RPV is now well established as another option for the treatment of HIV. Its monthly intramuscular administration has revolutionized the battle for patient medication compliance.

The initiation of CAB/RPV requires diagnosis of HIV-1 infection, no prior failures or resistance to CAB or RPV, a stable antiretroviral regimen, and viral suppression for 3 months with an understanding of monthly compliance.^5^ Adverse reactions include nausea, headache, anxiety, depression, hypersensitivity reactions, and hepatotoxicity. CAB/RPV is associated with ALT elevations in ∼7% of patients, but these are generally transient and rarely require dose modifications.^6,7^ CAB and RPV are not effective in treating HBV.^2^ Patients planning to transition from ART to CAB/RPV must be screened for HBV and receive their HBV vaccine with confirmation of immunity. If patients are infected with HBV or treated with ART, they are not eligible for initiation of CAB/RPV due to the possibility of an acute increase in HBV DNA.^8^

In a case report by Pintado et al,^9^ their patient also received his HBV vaccination, and CAB/RPV was initiated. Unlike our patient, however, anti-HBs levels were only checked after the patient tested positive for an acute HBV infection after CAB/RPV initiation. Our patient's anti-HBs levels confirmed immunity with a level greater than 10 mIU/mL (14 mIU/mL). Despite this, our patient still developed an acute HBV infection. It is important to note that our patient continued to participate in high-risk behavior after the switch to CAB/RPV. Though routine laboratory results were conducted monthly, HBV tests were not routinely done during follow-ups. To our knowledge, the case report by Pintado et al is the only other case report that provides an association with a patient with HIV, vaccinated from HBV, who developed an acute HBV infection after CAB/RPV treatment (Table 1). We searched for publications in PubMed from the last 30 years. Current guidelines state that CAB/RPV should not be started in patients with active HBV coinfection without concurrent oral therapy.^10^ There are currently no testing guidelines for HBV while patients are on long-acting injectable therapies.

**Table 1.** Comparison of cases of patients with HIV who were vaccinated before starting CAB/RPV

Case	Patient age	HIV	History of prior HBV infection	Vaccinated	Anti-HBs (IU/L)	HBsAg	Anti-HBc	Escape mutation identified	Developed acute HBV
Boateng et al	30	Yes	No	Yes	14.5	Negative	Not tested	Not tested	Yes
Pintado et al^9^	52	Yes	No	Yes	Not tested	Negative	Not tested	Not tested	Yes

Anti-HBc, hepatitis core antibodies; Anti-HBs, hepatitis B surface antibody; CAB, cabotegravir; HBsAg, hepatitis B surface antigen; HBV, hepatitis B virus; HIV, human immunodeficiency virus; RPV, rilpivirine.

Even with serologies indicating immunity, patients are still at risk of acute HBV infection. In patients who were on ART and who are switched to CAB/RPV, there are possibilities of HBV reactivation.^2,11^ Though rare, there are cases of occult HBV infection (OBI) (HBV infection in patients testing negative for HBsAg and serum HBV DNA) and escape mutations (HBV infection due to mutations in vaccinated patients) that put patients who are not on HBV therapy at risk of an acute infection.^2,12–14^

**Figure 2.**
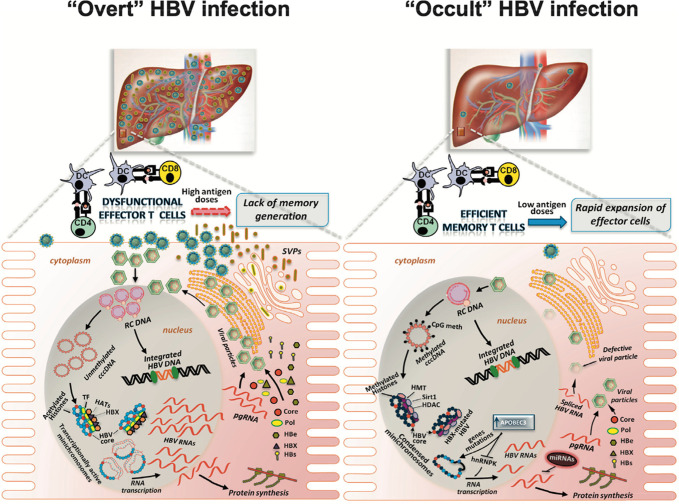
Comparison of overt HBV infection and occult HBV infection. Low levels of HBV DNA in occult HBV infection elicits a low antigen response. This will lead to low serum hepatitis B surface antigen, but hepatitis core antibodies will be present. APOBEC3, apo-B mRNA editing enzyme catalytic polypeptide; cccDNA, covalently closed circular DNA; CD4, CD4^+^ T cell; CD8, CD8^+^ T cell; CpG meth, methylated CpG islands; DC, dendritic cell; HATs, histone acetyltransferases; HBs, envelope proteins; HBV, hepatitis B virus; HMT, histone methyltransferases; hnRNP K, heterogeneous nuclear ribonucleoprotein K; miRNAs, microRNAs; pgRNA, pregenomic RNA; RC DNA, relaxed circular DNA; SVPs, subviral particles; TF, cellular transcription factors. Adapted from ref. 13.

OBI is due to significant suppression of HBV by the immune system, leading to low levels of HBV DNA and the absence of HBsAg (Figure 2).^12,13^ HBV DNA in OBI is generally found in low levels in the liver and serum (<200 IU/mL). It was found that most patients with OBI were positive for hepatitis core antibodies (anti-HBc).^2,12,13^ Forgetting to include anti-HBc in routine serology testing can lead to a missed HBV infection diagnosis in patients with HIV/HBV coinfection. OBI is commonly implicated in patients with underlying chronic hepatitis, HBV transmission through liver transplants, and perinatal transmission. Common ways to diagnose OBI include testing for anti-HBc and liver biopsy. Our patient was only tested for hepatitis B surface antibody (anti-HBs) and hepatitis B surface antigen due to his vaccination history. He was never tested for anti-HBc nor was a liver biopsy done, and therefore, an OBI cannot be ruled out.

Vaccination may be ineffective in patients with escape mutations due to antibodies that cannot recognize the mutated virus, leading to a breakthrough infection.^14^ Escape mutations are due to point mutations that alter the HBV surface antigen. Owing to these mutations of the surface gene (S-gene), neutralizing antibodies that develop from vaccination and exposure to hepatitis B immunoglobulins no longer recognize HBV, resulting in breakthrough infections.^14,15^ Identification of a possible HBV infection with escape mutation begins with the presence of HBsAg and anti-HBs, generally in vaccinated patients. The ideal method to identify these mutations is by analyzing the HBV DNA of the S-gene regions with nucleic acid amplification tests.^16,17^ Our patient was confirmed to be immunized with negative HBsAg and anti-HBs titers consistent with vaccination. Despite this, it is still challenging to exclude an HBV infection. Although rare, ongoing studies have associated OBI with specific escape mutations that cause negative HBsAg serologies.^17^ Without evaluating HBV DNA, we cannot conclude that our patient did not have an escape mutation.

In clinical practice, evaluating the HBV DNA for each patient who plans to switch to CAB/RPV may not be practical due to the cost of nucleic acid amplification test.^18^ The incidence of OBI and HBV with escape mutations is challenging to assess.^16,18^ It is essential to know that HBV infection is possible despite vaccination. In patients who want to switch to or are currently on CAB/RPV, physicians may need to discuss the need for supplemental HBV therapies with or without an active HBV infection. Before initiating CAB/RPV, patients should be screened for anti-HBc, anti-HBs, and HBsAg. We know that by checking for anti-HBc, we have a high likelihood of finding OBIs.^12,18^ While on treatment, patients should be regularly screened for HBV with anti-HBc, anti-HBs, and HBsAg. If there is high suspicion of HBV, testing for HBV DNA or a liver biopsy can be considered. Guidelines for monthly or bi-monthly HBV testing in patients on CAB/RPV may be beneficial, especially in patients who partake in high-risk behaviors.

In conclusion, the long-acting injectable formulation of CAB and RPV has been groundbreaking in the management of HIV by improving compliance with monthly administrations. Owing to the lack of HBV coverage, patients who switched from ART to CAB/RPV may be at risk of acute HBV infection. Vaccinated patients should not be stamped as low risk for HBV infection due to the possible presence of OBI and HBV escape mutations. Our case presents a unique occurrence of acute HBV infection in a vaccinated patient that portrays the risks that must be considered when using novel therapies. Screening for HBV is essential, with consideration of supplemental HBV therapies in high-risk patients. Guidelines may need to be established to involve routine HBV testing in patients on CAB/RPV.

## DISCLOSURES

Author contributions: W. Boateng: editing and creating the manuscript, and is the article guarantor. N. Carlin and E. Spira: editing and reviewing the manuscript. M. Szabela and K. Ezeh reviewed the manuscript.

Financial disclosure: None to report.

Informed consent was obtained for this case report.
